# Family-based clusters of cognitive test performance in familial schizophrenia

**DOI:** 10.1186/1471-244X-4-20

**Published:** 2004-07-22

**Authors:** Fabian Hoti, Annamari Tuulio-Henriksson, Jari Haukka, Timo Partonen, Lasse Holmström, Jouko Lönnqvist

**Affiliations:** 1 Department of Mathematics and Statistics, University of Helsinki, Finland; 2 Department of Mathematical Sciences, University of Oulu, Finland; 3 Department of Mental Health and Alcohol Research, National Public Health Institute of Finland, Helsinki, Finland

## Abstract

**Background:**

Cognitive traits derived from neuropsychological test data are considered to be potential endophenotypes of schizophrenia. Previously, these traits have been found to form a valid basis for clustering samples of schizophrenia patients into homogeneous subgroups. We set out to identify such clusters, but apart from previous studies, we included both schizophrenia patients and family members into the cluster analysis. The aim of the study was to detect family clusters with similar cognitive test performance.

**Methods:**

Test scores from 54 randomly selected families comprising at least two siblings with schizophrenia spectrum disorders, and at least two unaffected family members were included in a complete-linkage cluster analysis with interactive data visualization.

**Results:**

A well-performing, an impaired, and an intermediate family cluster emerged from the analysis. While the neuropsychological test scores differed significantly between the clusters, only minor differences were observed in the clinical variables.

**Conclusions:**

The visually aided clustering algorithm was successful in identifying family clusters comprising both schizophrenia patients and their relatives. The present classification method may serve as a basis for selecting phenotypically more homogeneous groups of families in subsequent genetic analyses.

## Background

Schizophrenia is a severe mental illness which tends to run in families. Moreover, schizophrenia is a complex disorder with multiple environmental as well as genetic predisposing effects. Previous studies have shown that many neuropsychological functions are impaired in schizophrenia patients, and, to a lesser degree, also in their unaffected relatives [1-3]. Consequently, the continuous traits derived from neuropsychological tests have been suggested as one type of endophenotypes of schizophrenia to be included in genetic analyses [4-9], for a review see Egan and Goldberg [10]. Identifying more homogeneous subgroups of families with a similar pattern of cognitive test performance would further refine the data to be included in these analyses.

Recently, cluster analysis of verbal learning and memory tests was used to divide patients with schizophrenia into subtypes. Categorization by these cognitive traits resulted in meaningful subgroups of schizophrenia [11]. In another study, extended neuropsychological test data of patients with schizophrenia were included in a hierarchical and iterative partitioning cluster analysis [12]. Four clusters were identified, ranging from good performance to profound global dysfunction. In Sautter et al. [13] an exploratory study comparing clustering of neuropsychological test performance in schizophrenia patients with familial history to those without was performed. In their analysis, patients with family history fell into three distinct clusters, while only one homogeneous cluster was found for the non-familial group. However, only patients were included in the analyses of these studies. As schizophrenia is likely to be a multifactorial disorder with low penetrance, the inclusion of relatives in the clustering analyses would be a powerful way to reveal subgroups based on the endophenotype of interest.

In the present study, we report a new visually aided clustering approach aimed at identifying clusters of multiply affected families with schizophrenia on the basis of performance in neuropsychological tests. In the clustering process, each family was represented by the test scores of its affected and unaffected members, and the closeness the families was defined by the maximum pairwise distance between the members of the families. To our knowledge, this is the first study in which the clustering has been applied to families instead of solely to affected subjects with schizophrenia.

## Methods

### Subjects and data collection

From a general population cohort of people born between 1940 and 1976 inclusive in Finland, a northern European country with approximately 5 million inhabitants, we identified 33,731 individuals with a diagnosis of schizophrenia, schizoaffective disorder or schizophreniform disorder. Data on the diagnosis were derived from three nation-wide computerized health care registers covering the years 1969 to 1998: the Hospital Discharge Register, the Free Medicine Register, and the Pension Register. Linking the personal identification numbers of the affected subjects to the National Population Register database allowed us to identify their family members and to construct pedigrees.

Information on families with at least two members with schizophrenia, schizoaffective disorder or schizophreniform disorder, and at least two members with no diagnosis of psychiatric disorder was received from the aforementioned registers for 895 families from the whole of Finland. A blood sample for subsequent genetic analyses was drawn from 2295 subjects of 643 families. All available case note records were collected for those with a diagnosis of schizophrenia, schizoaffective disorder or schizophreniform disorder in any of the three registers. Two psychiatrists independently assessed the lifetime diagnoses for each case, according to the Diagnostic and Statistical Manual of Mental Disorders (DSM-IV) [14]. One of the assessors also completed the Operational Criteria Checklist for Psychotic Illness (OPCRIT) [15]. The collection of blood samples complied with the Declaration of Helsinki and its amendments. The protocol was accepted by the Ethics Committee of the National Public Health Institute, and the study was approved by the Ministry of Social Affairs and Health.

Of those multiply affected families who already had given the blood samples, a subsample was targeted for collection of more detailed phenotypic information. This sample was selected randomly based on the data from the registers and the OPCRIT process. All subjects from the families gave a written informed consent for the study protocol comprising a diagnostic research interview and neuropsychological testing. Both patients and their family members were interviewed using the Structured Clinical Interview for DSM-IV (SCID-I for axis I disorders and SCID-II for axis II disorders) [16]. All the interviewers were trained in a similar manner for the use of these instruments. The final consensus diagnoses were based on the data collected from the records, the OPCRIT process, and the SCID interview. A total of 281 subjects from 54 families fulfilled the inclusion criteria and thus included at least two siblings with schizophrenia, schizoaffective disorder or schizophreniform disorder, and at least two siblings without these disorders. Altogether 16 patients were excluded because of being too psychotic (*n = *6), having a current substance use diagnosis (*n *= 6), or being mentally retarded (*n *= 4). Of the family members to whom no psychiatric diagnosis was assigned for their lifetime, 6 were excluded because of high age, or for a defect in vision or hearing. The final sample thus comprised 165 subjects with a psychiatric diagnosis and 94 unaffected family members from 54 families. Of the 165 subjects with a diagnosis, altogether 82 subjects had schizophrenia, while 13 subjects suffered from schizoaffective disorder, 10 from schizophreniform disorder and 12 from bipolar disorder. A nonpsychotic disorder was assigned to 48 individuals. The 94 unaffected subjects did not get any current or lifetime psychiatric diagnosis. In 51 families, at least one of the patients included in the analysis suffered from pure schizophrenia. In the remaining three families, at least one subject with schizoaffective or schizophreniform disorder was included. All families from which the subjects for the present study were drawn, represent familial schizophrenia, as in each of them there were at least one sibling with a diagnosis of pure schizophrenia, plus at least one other sibling with schizophrenia, schizoaffective disorder or schizophreniform disorder.

### Test procedures

A neuropsychological test battery was administered to all subjects in fixed order by well-trained examiners either after the interview during the same day, or the following day. All examiners were psychologists or advanced psychiatric nurses extensively trained and supervised with the test battery. Experienced psychologists scored all the tests.

Auditory attention was assessed with the Digit Span Forward task, and verbal working memory with the Digit Span Backward task of the Wechsler Memory Scale-Revised (WMS-R) [17]. According to Finnish normative data, the test-retest reliability coefficients of the Span subtests vary with age from 0.74 to 0.82 [18].

The Visual Span forward subtest of the WMS-R [17] was used to assess visual attention. The backward condition of the span task was used for measuring visual working memory. According to Finnish normative data, the test-retest reliability coefficients of the Visual Span subtests vary with age from 0.72 to 0.80 [18]. The Logical Memory story A, of the WMS-R [17], immediate and delayed, was used to assess recall and retention in a story format. Visual memory was measured by the Visual Reproduction subtest of the WMS-R [17], immediate and delayed. In Finnish normative data, the test-retest reliabilities of these subtests have varied with age from 0.84 to 0.91, and 0.31 to 0.34, respectively.

Verbal learning and memory were assessed with the California Verbal Learning Test (CVLT) [19] which examines recall and recognition of word lists over a number of trials. The present study reports the following variables derived from the test: verbal learning (total recall over 5 trials), semantic clustering, and recognition memory (discriminability). No reliability data for Finnish subjects exist, but the split-half reliability of the CVLT is 0.77 to 0.86, according to the test manual [19].

Controlled Oral Word Association test (COWA) [20] was used to assess verbal fluency. The quantity of words the subject produces in one minute, both with words beginning with a designated letter (S,K), and within a category (animals), was assessed. No reliability data for Finnish subjects are available.

Four subtests of the Wechsler Adult Intelligence Test – Revised (WAIS-R) [21] were used. Verbal abilities were measured with the Vocabulary and Similarities subtests. Vocabulary is considered the best single measure of general ability [22]. The Similarities subtest is a task of abstraction and concept formation. The Block Design and Digit Symbol subtests have a motor component as the trials are timed. The former is a measure of visuospatial reasoning and abstraction. The latter subtest measures psychomotor performance. According to Finnish normative data, the test-retest reliabilities for Vocabulary, Similarities, Block Design, and Digit Symbol are 0.89–0.95, 0.69–0.88, 0.78–0.83, and 0.82–0.86, respectively, depending on age [23].

### Clustering and statistical analyses

#### Notation and imputation of missing values

The variables used in cluster analysis included 17 neuropsychological test variables together with the age and the sex of the subjects. With a total of *M *= 19 variables and *N *= 259 subjects, the data formed an *M *× *N *matrix *x *= (*x*_*ik*_), where *x*_*ik *_is the the value of the *i*th variable for the *k*th subject. However, there were 85 (1.7 %) missing values as not all test results were obtained for all subjects. The missing values were handled by the following procedure, which replaces an individual's missing value with an estimate obtained from a linear fit between the test with the missing value and the test that correlates with it most and that also has the individual's test result available.

1. Pairwise correlations were calculated between all test variables using only subjects with results available for both tests. We denote such correlation between the tests *i *and *j *by c_*ij*_.

2. Given a missing value in the test *i *for the subject *k*, we found the test *j *= *j*_0 _which had the highest value of |c_*ij*_| among the tests with the value *x*_*jk *_available and set



where the coefficients a and *b *were found by computing linear regression of the test *j*_0 _on the test *i *using only subjects with results available for both tests.

#### Cluster analysis

The families were clustered using a complete-linkage clustering algorithm. Each variable was normalized by subtracting the mean value and dividing by the standard deviation. The normalization was done to ensure that each variable contributes equally to the clustering procedure. Denote by *x*_*k *_= (*x*_1*k*_,...,*x*_*Mk*_) the normalized data for subject *k *and define the distance between two clusters *C*_*r *_and *C*_*s *_by

*d*_*rs *_= max{||*x*_*k *_- *x*_*l*_|| : *x*_*k *_∈ *C*_*r *_and *x*_*l *_∈ *C*_*s*_},

that is, *d*_*rs *_is the maximum pairwise distance between members of the two clusters. Here ||·|| denotes the euclidean distance, . In the sense of this distance measure, two clusters are close when all subjects in both clusters are close.

Clustering was carried out using the following algorithm.

1. Initial clusters are defined by the families.

2. The two clusters with the smallest inter-cluster distance *d*_*rs *_are merged into one larger cluster.

3. Steps 2 and 3 are repeated until a desired number of clusters remains.

**Figure 1:**
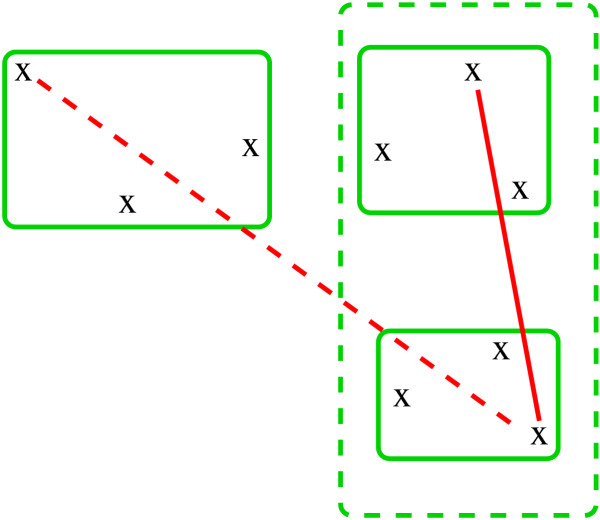
**Visualization of clustering. **Two merging steps of the clustering algorithm (see the text).

In Figure 1, two steps of the above procedure are demonstrated. Three clusters are depicted by the green solid lines. The two nearest clusters are combined (the dashed green line). Their inter-cluster distance *d*_*rs *_is shown by the solid red line. The inter-cluster distance between the two remaining clusters is shown by the dashed red line. Note that by using a different inter-cluster distance measure, such as the minimum pairwise distance, a different merging order would result (see the Discussion).

#### Visualization of clusters

**Figure 2:**
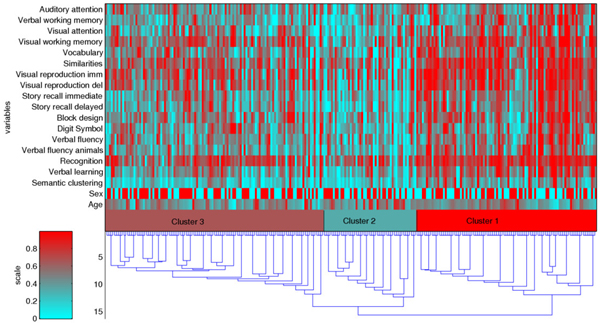
**Visualization of clustering result. **A visualization of the data and the cluster solution using the data image and the dendrogram. On the last line of the data image clusters 1, 2 and 3 are depicted with different colors. Both the subjects and the neuropsychological tests were ordered by a complete linkage clustering algorithm (see the text).

We introduce a visualization technique that helps in identifying candidate clusters and also gives an overall picture of the main differences between the produced clusters as measured by all variables simultaneously. The method gives information about the dynamics of the clustering process and the characteristics of the candidate clusters. The upper part of Figure 2 presents the data matrix as what is called the "color histogram" [24] or "the data image" [25]. The rows correspond to variables and the columns correspond to subjects. The values of the neuropsychological tests and other variables are visualized using color coding. The color of a variable changes from blue to red as its value increases. To better utilize the dynamic range of coloring, 5 % of the highest values and 5 % of the lowest values were set to the 95th percentile and the 5th percentile of the test results, respectively. The variables were then shifted and scaled to the interval [0,1] and color coded (0 = blue, 1 = red). On the last line of the color histogram, clusters are depicted with different colors.

To further improve the visual impression of the clusters, the neuropsychological test variables (the rows of the data image) were ordered using essentially the same procedure that was used in clustering the families. The initial clusters were now the individual variable vectors *x*_*i *_= (*x*_*i*1_,...,*x*_*iN*_) and the pairwise distance between two clusters *C*_*r *_and *C*_*s *_was defined as

*d*_*rs *_= max{1/|*c*_*ij*_| : *i *∈ *C*_*r*_, *j *∈ *C*_*s*_},

where *c*_*ij *_denotes the correlation between the variables *i *and *j*. Thus, at each step, the algorithm merged clusters with the highest correlating variables.

**Figure 3:**
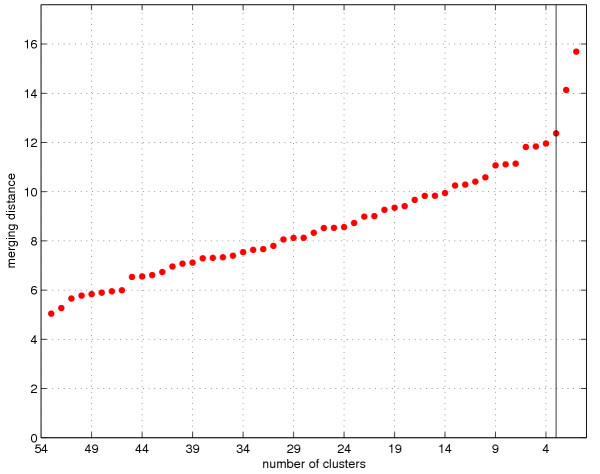
**Merging distances. **The inter-cluster distance (merging distances) as a function of the number of clusters. The vertical line indicates the suggested three cluster result, after which there is a clear jump in the merging distances.

The lower part of Figure 2 visualizes the actual clustering process using the dendrogram. The history (vertical direction) of the mergings is shown from the beginning (one family in each cluster) to the end (all families in one cluster). By simultaneously exploring the two images, a reasonable value for the number of clusters can be found and the characteristics of the cluster solution visualized in a useful manner. It is also helpful to monitor the inter-cluster distance measure for possible large jumps which indicate that two distant clusters are being merged (Figure 3).

#### Validation of cluster result

The clusters were obtained by treating families as single objects whose dissimilarity was measured by the pairwise test performance differences between the family members. One may therefore ask whether the clusters found still appear to be distinct groups when viewed simply as sets of individual subjects. We examined this question by dividing repeatedly the 54 families into three random clusters that had the same number of families as in the proposed three cluster solution and by computing, for each of the three pairs of the generated clusters, the ratio *BW*_*r*,*s *_= *B*_*r*,*s*_/(*W*_*s *_+ *W*_*r*_), where *B*_*r*,*s *_is the mean distance between subjects from clusters *r *and *s *(in the 19-dimensional space) and *W*_*r *_is the mean distance between subjects within cluster *r*. The statistic *BW*_*r*,*s *_takes on a large value if the distance between the subjects from the different clusters is large compared to the distance between the subjects within the clusters themselves indicating that the two clusters are separated in the 19-dimensional space defined by the variables used. If the values of *BW*_*r*,*s *_for the proposed three clusters are significantly higher than for a random partition we take this as evidence that the clusters found indeed constitute meaningful groups also at the level of individual subjects.

Further, after the cluster analysis, the proposed family clusters were examined for differences on demographic and neuropsychological measures. In addition, the patients included in the clusters were examined for the differences in clinical variables as evaluated by the OPCRIT (premorbid social adaptation, response to neuroleptic treatment, chronicity, age of onset) of the disorder. In comparing the demographic and clinical variables, the Chi-square test, or t-test, both two-tailed, were applied. The differences in the quantitative neuropsychological measures were analyzed using the linear mixed effects (LME) model, which takes into account the dependence between the subjects, who, *a priori, *came from the same families. Thus, family was included as a random effect in all models with age and sex as the fixed effects. In addition, post hoc models were conducted with education years as an added fixed effect, a known confounder for cognitive functions. In all these analyses, the probability level < 0.05 indicated statistical significance. Analyses were performed using the S-Plus statistical software, version 3.4 [26].

## Results

### The cluster solution

Three clusters of families were successfully identified from the study sample. The first cluster comprised 94 subjects from 17 families, the second cluster 50 subjects from 12 families, and the third cluster 115 and 25. Adding more neuropsychological test variables or leaving out the sex or the age of the subjects had little effect on the solution.

The data image (Figure 2) indicated that the overall performance of the subjects was higher in the first cluster than in the second, and that the performance in the third cluster was between the other two. The three clusters were therefore identified as consisting of subjects that were relatively well-performing, impaired and intermediate, respectively.

A three cluster solution is supported by the homogeneity of the within-cluster test performance patterns of the proposed groups (Figure 2). As shown by the dendrogram, the two-cluster solution would combine the impaired and the intermediate clusters, and the four-cluster result would divide the well-performing cluster into two subclusters one of which is very small, consisting only of six families. Stopping the merging process even earlier does not appear to suggest any interesting alternative cluster solutions. Note also the jump in the distance function of Figure 3 after 3 clusters.

**Figure 4:**
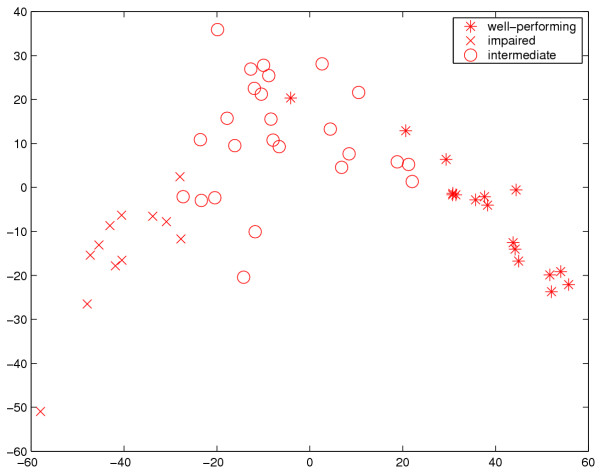
**Multidimensional scaling visualization. **A two-dimensional visualization using multidimensional scaling (MDS) of the families in the three clusters found. The similarity measure employed in MDS was the same one that was used in the family clustering procedure, with the natural modification that the distance between a family and itself was set to zero. The horizontal and vertical axes are the directions with the highest and the second highest variance, respectively.

In Figure 4 the three family clusters are further visualized by classic metric multidimensional scaling (MDS) [27,28]. Thus, with a total of 19 variables, the 54 families comprising the three clusters are represented as points in the 19-dimensional euclidean space so that the pairwise distances between the points match the original distances between the families (maximum pairwise euclidean distances between subjects in the families). The two-dimensional projection of the 19-dimensional space, although capturing only 27.0% of the total variation, shows the two most important directions (the directions with the highest variance) and provides evidence on the success of the clustering process itself, i.e., in the sense of the distance measured used, clustering does appear to produce three separate classes of families. Further visualization of the clusters, including an animation, is provided by the supplementary material to this article (See Additional file 1, 2, 3, 4 and 5.

In each of the three pairwise comparisons of the statistic *BW*_*r*,*s *_the randomly generated clustering solution almost always had a smaller value than the proposed clustering solution (the fraction of opposite results in 10 000 trials was less than 0.01). This lends support to the visual impression that the three clusters are separate groups when viewed as subsets of individual subjects. Results were similar when the family structure was ignored and the random clusters were generated allowing subjects from the same family to be assigned to different clusters.

### Demographic and clinical characteristics

The demographic characteristics of the clusters of families are shown in Table 1, and Table 2 shows the clinical characteristics of the subjects with schizophrenia, schizoaffective disorder or schizophreniform disorder. The three clusters did not differ by age or sex distribution. The well-performing cluster had significantly more years of education than the two others (*p <*0.001 in contrasts versus both other clusters). Overall, the clusters did not differ in clinical characteristics, except that the well-performing cluster showed better premorbid adaptation than the intermediate cluster (p = 0.04). The age of onset did not differ between the clusters (mean 25.9, SD 7.8, mean 24.7, SD 7.6, mean 23.7, SD 7.6 in clusters 1, 2, and 3, respectively, all *p*-values > 0.20). The impaired cluster did not include any patients with schizoaffective disorder, bipolar disorder or other affective psychotic disorders, while in the well-performing and intermediate clusters, these diagnoses were assigned to 14% and 11% of the subjects, respectively. About 36% of family members in all three clusters were unaffected.

**Table 1:** Demographic characteristics Demographic characteristics of the family members in the well-performing (Cluster 1), impaired (Cluster 2), and intermediate (Cluster 3) clusters.

	Cluster 1 (*n *= 94, 17 families)		Cluster 2 (*n *= 50, 12 families)		Cluster 3 (*n *= 115, 25 families)	
	Mean	SD	Mean	SD	Mean	SD
Sex (F/M)	48/46		24/26		50/65	
Age	48.8	9.4	52.3	12.6	48.5	11.3
Education years	11.3^*a*,*b*^	3.0	9.3	2.4	9.9	2.3

^*a*^Contrast versus cluster 1 and 2, *p *< 0.001, t-test, two-tailed. ^*b*^Contrast versus cluster 1 and 3, *p *< 0.001, t-test, two-tailed.

**Table 2:** Clinical characteristics Clinical characteristics of the affected family members in the well-performing (Cluster 1), impaired (Cluster 2), and intermediate (Cluster 3) clusters

	Cluster 1 (*n *= 35)		Cluster 2 (*n *= 27)		Cluster 3 (*n *= 43)		*p*		
	Yes	No	Yes	No	Yes	No	1 vs. 2 *	1 vs. 3*	2 vs. 3*
Poor premorbid social adjustment	14	21	13	14	27	16	ns	0.04	ns
Response to neuroleptics	30	5	21	6	34	9	ns	ns	ns
Chronic course of the disorder	15	20	17	10	24	19	ns	ns	ns

* Chi square, two-tailed.

### Neuropsychological variables

The impaired cluster scored lowest in all measured neuropsychological variables, and the intermediate cluster showed consistently worse performance than the well-performing one (Table 3). The differences between the family clusters in the neuropsychological variables were tested by the within-family linear mixed effect models. In these models, the impaired cluster was found to achieve significantly lower scores than both other clusters in almost all traits (Table 4). The only variable not reaching statistical significance in differentiating any of the clusters was auditory attention.

**Table 3:** Neuropsychological test performance Means and Standard Deviations in neuropsychological test performance (raw scores) among the family members in the well-performing (Cluster 1), impaired (Cluster 2), and intermediate (Cluster 3) clusters

	Cluster 1 (*n *= 94)		Cluster 2 (*n *= 50)		Cluster 3 (*n *= 115)	
	Mean	SD	Mean	SD	Mean	SD
Auditory attention	6.7	2.1	5.8	1.9	6.3	1.8
Verbal working memory	6.1	2.3	4.5	2.0	4.7	1.7
Visual attention	8.6	2.1	6.8	1.9	7.6	1.8
Visual working memory	7.8	2.1	5.3	2.7	6.9	2.0
Story recall immediate	20.1	7.9	11.7	6.8	14.5	7.1
Story recall delayed	16.6	8.0	8.0	6.9	11.2	6.8
Visual reproduction imm	32.1	6.9	23.5	11.4	27.6	8.4
Visual reproduction del	27.4	9.9	16.2	12.8	21.2	10.5
Vocabulary	41.8	12.1	27.8	14.3	33.1	11.6
Similarities	24.7	4.6	18.5	6.7	21.1	5.3
Digit Symbol	39.4	16.5	26.9	13.6	34.4	13.8
Block design	28.6	11.6	17.7	12.5	22.3	10.8
Verbal learning	45.2	12.2	30.9	11.5	36.6	11.7
Semantic clustering	13.2	8.4	6.7	4.8	8.1	6.6
Recognition	93.2	6.0	81.1	16.6	87.2	10.3
Verbal fluency	29.9	11.5	22.0	10.4	25.2	9.3
Verbal fluency, animals	20.2	6.2	14.2	5.2	16.2	5.0

**Table 4:** Differences in neuropsychological test performance Differences in neuropsychological test performance between the well-performing (Cluster 1), impaired (Cluster 2), and intermediate (Cluster 3) clusters. Linear mixed effects models with family as a random effect, and sex and age as fixed effects

	Cluster 2 vs. Cluster 1				Cluster 1 vs. Cluster 3				Cluster 2 vs. Cluster 3			
	Coeff	SD	Wald	*p*	Coeff	SD	Wald	*p*	Coeff	SD	Wald	*p*
Auditory attention	-0.81	0.43	-1.87	0.07	0.40	0.35	1.12	0.28	-0.41	0.41	-1.00	0.32
Verbal working m	-1.52	0.46	-3.31	0.002	1.40	0.38	3.74	< 0.001	-0.11	0.44	-0.27	0.80
Visual attention	-1.65	0.39	-4.23	< 0.001	1.00	0.31	3.19	0.002	-0.65	0.37	-1.74	0.08
Visual working m	-2.31	0.46	-4.98	< 0.001	0.91	0.38	2.42	0.02	-1.40	0.44	-3.17	0.003
Story recall imm	-8.18	1.65	-4.95	< 0.001	5.71	1.33	4.29	< 0.001	-2.47	1.56	-1.58	0.11
Story recall del	-8.38	1.59	-5.27	< 0.001	5.37	1.28	4.20	< 0.001	-3.01	1.50	-2.00	0.05
Visual reprod imm	-7.82	1.56	-5.02	< 0.001	4.49	1.23	3.65	< 0.001	-3.33	1.49	-2.23	0.03
Visual reprod del	-10.20	2.03	-5.03	< 0.001	5.93	1.60	3.71	< 0.001	-4.26	1.93	-2.21	0.03
Vocabulary	-14.39	2.39	-6.03	< 0.001	8.64	1.92	4.50	< 0.001	-5.74	2.29	-2.51	0.02
Similarities	-9.95	2.43	-4.09	< 0.001	3.51	0.84	4.17	< 0.001	-2.45	1.00	-2.45	0.02
Digit Symbol	-10.01	2.66	-3.91	< 0.001	4.42	2.13	2.08	0.04	-5.66	2.54	-2.23	0.03
Block design	-8.48	2.76	-3.07	< 0.004	6.64	1.96	3.38	0.001	-3.31	2.31	-1.43	0.16
Verbal learning	-13.74	2.11	-6.53	< 0.001	8.22	1.69	4.87	< 0.001	-5.52	2.03	-2.72	0.009
Semantic clust	-6.20	1.21	-5.12	< 0.001	4.92	0.97	5.10	< 0.001	-1.28	1.17	-1.09	0.28
Recognition	-11.21	1.82	-6.14	< 0.001	5.79	1.45	3.98	< 0.001	-5.41	1.77	-3.07	0.004
Verbal flu	-7.50	2.21	-3.40	0.001	4.59	1.79	2.57	0.01	-2.90	2.08	-1.40	0.17
Verbal flu, anim	-5.80	1.00	-5.78	< 0.001	4.01	0.80	5.03	< 0.001	-1.79	0.96	-1.87	0.07

### Effect of education

As the clusters differed significantly from each other in education years, we conducted post hoc linear mixed effects models with family as the fixed effect, and age, sex and education years as the random effects (data not shown). This did not eliminate the significant differences in cognitive functioning between the well-performing and the impaired cluster. In contrasts between the well-performing and intermediate cluster, all other differences remained significant, except in the scores of Visual immediate recall, Digit Symbol, and Verbal fluency, which lost their significance. Between the intermediate and the impaired cluster, scores in Vocabulary and Digit Symbol were no longer significantly different after controlling for education years.

## Discussion

We report on the application of a visually aided clustering algorithm to data based on performance in a set of neuropsychological test measures, these being potential endophenotypic traits in schizophrenia. We were able to successfully detect three separate family clusters comprising both schizophrenia patients and their family members. In the impaired cluster, the families scored significantly worse than those in the other two. The well-performing cluster received the highest scores in each cognitive test, and the intermediate cluster scored consistently between the other two. However, the clusters of families did not differ from each other in age, sex distribution, and, regarding the affected subjects, in the age of onset, or in most of the other clinical features. The well-performing cluster was significantly more educated than the two others, but controlling for education years did not change the main results.

We tested the differences in the diagnostic class distributions (including those with no diagnosis), and although the differences did not reach statistical significance, we find it interesting that none of the subjects with schizoaffective disorder, bipolar disorder, or other affective psychotic disorders ended up into the impaired cluster. We consider this as supporting the validity of particularly the poor cluster, which seems to represent a subsample of core schizophrenia with the most defected cognitive functioning. This cluster included the same proportion of unaffected subjects than the other two clusters, and based on the clustering algorithm, these family members without any psychiatric diagnoses during their lifetime performed generally poorly, too.

Global verbal memory, including the story recall from the WMS-R [17] and verbal learning from the CVLT [19], were among the measures that differentiated well the clusters. This is in line with results by Heinrichs and Zakzanis [29], who found the best effect sizes in these functions in differentiating schizophrenia patients from controls. However, against a background of global dysfunction, any selective impairments such as those in verbal memory, are only relative [29]. The present study suggests that it is possible to characterize families with convergent cognitive performance using variables from several domains of cognition, such as attention, verbal memory, executive functioning, and intelligence. In efforts aiming at sample homogeneity, the best method may be using multiple endophenotypic measures. In part, our results are also comparable to those by Erlenmeyer-Kimling et al [30], who found that impairments in multiple cognitive measures best predicted future schizophrenia in high risk subjects.

Our results suggest that molecular genetic analyses could benefit from prior appliance of our method, revealing meaningful family subgroups in a representative sample of familial schizophrenia. It would allow the resources to be targeted primarily for gene hunting projects among more homogeneous groups of families. Our new approach to combining data visualization and clustering appears to offer a valuable tool for identifying clusters in family-based data. Applying hierarchical clustering and the data image interactively helps to identify a reasonable value for the number of clusters in the cluster solution. By ordering the variables in the data image suitably, one gains useful insight into the test performance characteristics of the subjects in the clusters.

As suggested in Palmer et al. [31], there may be a group of schizophrenia patients with no observed global impairment in cognition. One result of the present cluster analysis was the detection of a group of schizophrenia families with clearly better performance than the families in the two other clusters. This finding, together with those of previous studies, warrants further research for detecting putative factors protecting the cognitive development of these patients and their family members. Interestingly, attention, as measured by the simple auditory attention task (verbal span forwards), did not differentiate the clusters. The mean score in this task was also below the national normative mean in all clusters. This may indicate a fundamental impairment of attention in schizophrenia [32], observed also in patients and family members who otherwise perform well. When education was controlled for, it was further found that score in Digit Symbol, a test measuring information processing speed, and verbal fluency, a measure of executive function, did not any more separate the clusters. These results are in line with those in Weickert et al. [33], who found a selective impairment in executive function and attention in a group of schizophrenia patients defined as cognitively preserved.

The present study is the first one in which cluster analysis of neuropsychological test variables has been conducted among a representative sample of familial schizophrenia comprising both affected and unaffected family members. The sample of the present study was randomly selected from a nationwide familial schizophrenia cohort. However, the results may not be generalizable to families with only one patient with the disorder. The similar patterns in neuropsychological performance in the clusters may be due to a variety of familial environmental effects, which are difficult to define *ex post facto. *Furthermore, our set of neuropsychological measures did not cover all those cognitive domains that previous studies have suggested as valid cognitive endophenotypes. However, it has been demonstrated in twin and in family studies [3,7,34], that the cognitive traits from our test selection measuring attention, working memory, verbal memory and visual memory do show genetic effects. Furthermore, in the present sample, the included test variables discriminated the affected and unaffected subjects, both in the whole sample and within clusters (data not shown).

In the absence of a control sample, the present study could not test the possibility that the same clustering solution would emerge in normal families from the population. However, to our knowledge, such family clustering studies have not been conducted. In a study by Horan and Goldstein [35], a cluster analysis was conducted both in a patient and in a non-psychotic patient control group. The clustering solutions in these groups did not resemble each other, suggesting a specific pattern in the schizophrenic population. It is known that family members of schizophrenia patients tend to perform worse than subjects from control populations [1-3], and particularly those in multiply affected families [8,34]. Indeed, the aim of the present study was to explore the clustering of families in multiply affected families with schizophrenia. Thus the generalizability of the results may be limited to such samples representing about one fifth of all schizophrenia cases [36].

Clearly, the choice of the inter-cluster distance measure can greatly influence the merging process and hence the cluster solutions obtained. The maximum pairwise distance between subjects adopted in our analysis assigns a small distance between clusters only if all subjects in the clusters are close to each other in their test performance. We also experimented with the minimum pairwise distance but the results were poor. An explanation for this emerges by studying the minimum distance measure along the first few principal component directions of the normalized test results (the directions of largest variance). It turns out that, along these directions, most families have a member with nearly average performance and whose test results therefore closely match those of many members of other families. The variance of the distribution of the pairwise minimum distances is small and modest changes in the test results can lead to significantly different cluster solutions. The mean of pairwise distances between two clusters would be a compromise between the maximum and the minimum distances, but it turned out to behave much like the minimum distance and was therefore not used.

## Conclusions

The new approach which combines clustering and data visualization was effective in identifying homogeneous subgroups of schizophrenia families with convergent cognitive test performance. Our results emerging from a sample of familial schizophrenia patients are in line with previous studies in which two extreme clusters have consistently emerged, characterized by a well-performing and a dysfunctional group of subjects, and at least one intermediate [11,12,37]. Our results agree with those in Sautter et al. [13], in which neuropsychological data of familial schizophrenia patients formed three clusters with respect to the level of performance. The fact that our findings, after including both affected and unaffected subjects agree with prior evidence, suggest further use of the cognitive traits as valid endophenotypes to be used in genetic linkage analyses. This method seems valid for partitioning the schizophrenia families by a relevant phenotypic category, resulting in more homogeneous subgroups. The method and results of the present study may be exploited in selecting whole families for subsequent analyses using the actual genetic marker data.

## Competing interests

None declared.

## Authors' contributions

FH and LH made the cluster analysis and wrote the corresponding sections, ATH supervised the neuropsychological test data collection, performed the differences testing analyses and drafted the manuscript, TP was the clinical and diagnostic study leader and commented on the text, JH designed the models for differences testing, JL conceived the research project.

## Pre-publication history

The pre-publication history for this paper can be accessed here:



## Supplementary Material

Additional file 1 Comparison of Cluster 1 (well-performing) and Cluster 2 (impaired). The original variables (neuropsychological test variables + age) are visualized using the parallel coordinate plot. The subjects classified as well-performing are colored green and those classified as impaired are colored red. Yellow indicates overlap of green and red. There is overlap in all the original variables. The above figure was produced using the Crystal Vision software. The software uses a so-called grand tour technique to systematically (and in a continous manner) go through all possible rotations in the data space. The tour can be visually monitored using the parallel coordinate plot. Click here for file

Additional file 2 Smaller version of Additional file 1. Comparison of Cluster 1 (well-performing) and Cluster 2 (impaired). The original variables (neuropsychological test variables + age) are visualized using the parallel coordinate plot. The subjects classified as well-performing are colored green and those classified as impaired are colored red. Yellow indicates overlap of green and red. There is overlap in all the original variables. The above figure was produced using the Crystal Vision software. The software uses a so-called grand tour technique to systematically (and in a continous manner) go through all possible rotations in the data space. The tour can be visually monitored using the parallel coordinate plot. Click here for file

Additional file 3 A parallel coordinate plot of the data after a rotation transformation in the data space. The axes correspond now to linear combinations of the original variables. The 6th axis from the top shows an interesting one-dimensional projection of the data. In this direction the two clusters are well separated. The above figure was produced using the Crystal Vision software. The software uses a so-called grand tour technique to systematically (and in a continous manner) go through all possible rotations in the data space. The tour can be visually monitored using the parallel coordinate plot. Click here for file

Additional file 4 Smaller version of Additional file 3. A parallel coordinate plot of the data after a rotation transformation in the data space. The axes correspond now to linear combinations of the original variables. The 6th axis from the top shows an interesting one-dimensional projection of the data. In this direction the two clusters are well separated. The above figure was produced using the Crystal Vision software. The software uses a so-called grand tour technique to systematically (and in a continous manner) go through all possible rotations in the data space. The tour can be visually monitored using the parallel coordinate plot. Click here for file

Additional file 5 A movie which demonstrates the use of the software can be downloaded here. Smaller resolution CrystalVision.mpg(0.86 Mb). A higer resolution version of this movie is available at  Reference for Crystal Vision software CrystalVision, copyright (c) 2000 by Crystal Data Technologies (Qiang Luo, Edward J. Wegman, and Xiaodong Fu), is a Windows 95/98/NT package for Wintel computers. A demonstration version of CrystalVision is available at Click here for file
