# Supplementary figures and images for: Family-based clusters of cognitive test performance in familial schizophrenia
Source: BMC Psychiatry. 2004 Jul 22;4:20. doi: 10.1186/1471-244X-4-20 (PMC512293; doi:10.1186/1471-244X-4-20)

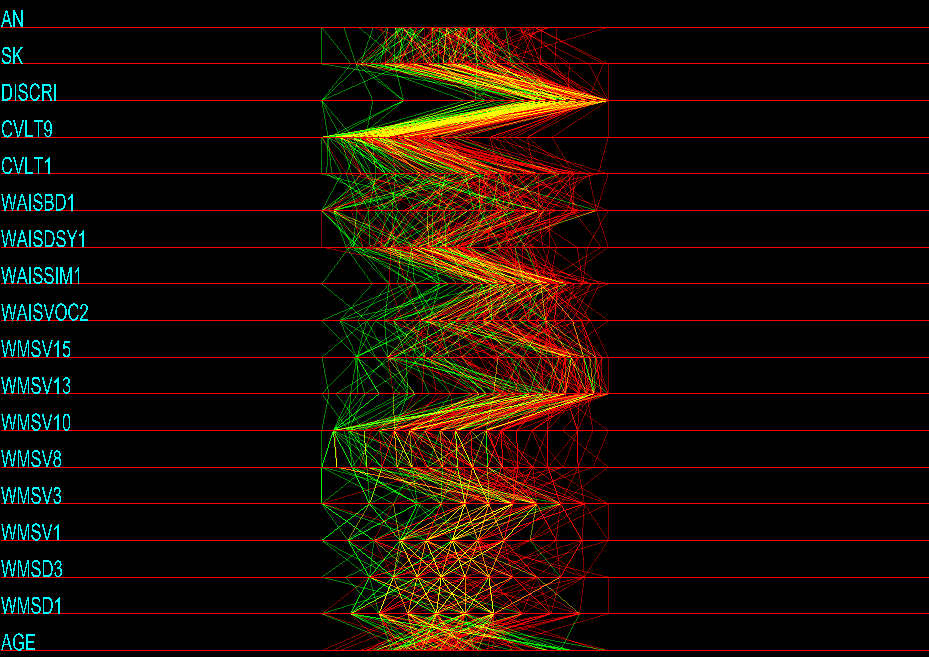

Supplement: Additional file 1 — Comparison of Cluster 1 (well-performing) and Cluster 2 (impaired). The original variables (neuropsychological test variables + age) are visualized using the parallel coordinate plot. The subjects classified as well-performing are colored green and those classified as impaired are colored red. Yellow indicates overlap of green and red. There is overlap in all the original variables. The above figure was produced using the Crystal Vision software. The software uses a so-called grand tour technique to systematically (and in a continous manner) go through all possible rotations in the data space. The tour can be visually monitored using the parallel coordinate plot. [file 1471-244X-4-20-S1.gif]

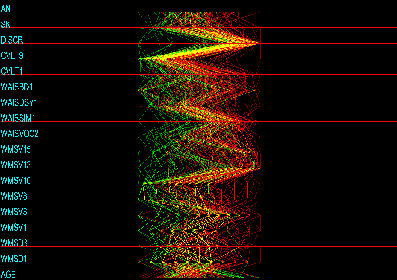

Supplement: Additional file 2 — Smaller version of Additional file 1. Comparison of Cluster 1 (well-performing) and Cluster 2 (impaired). The original variables (neuropsychological test variables + age) are visualized using the parallel coordinate plot. The subjects classified as well-performing are colored green and those classified as impaired are colored red. Yellow indicates overlap of green and red. There is overlap in all the original variables. The above figure was produced using the Crystal Vision software. The software uses a so-called grand tour technique to systematically (and in a continous manner) go through all possible rotations in the data space. The tour can be visually monitored using the parallel coordinate plot. [file 1471-244X-4-20-S2.gif]

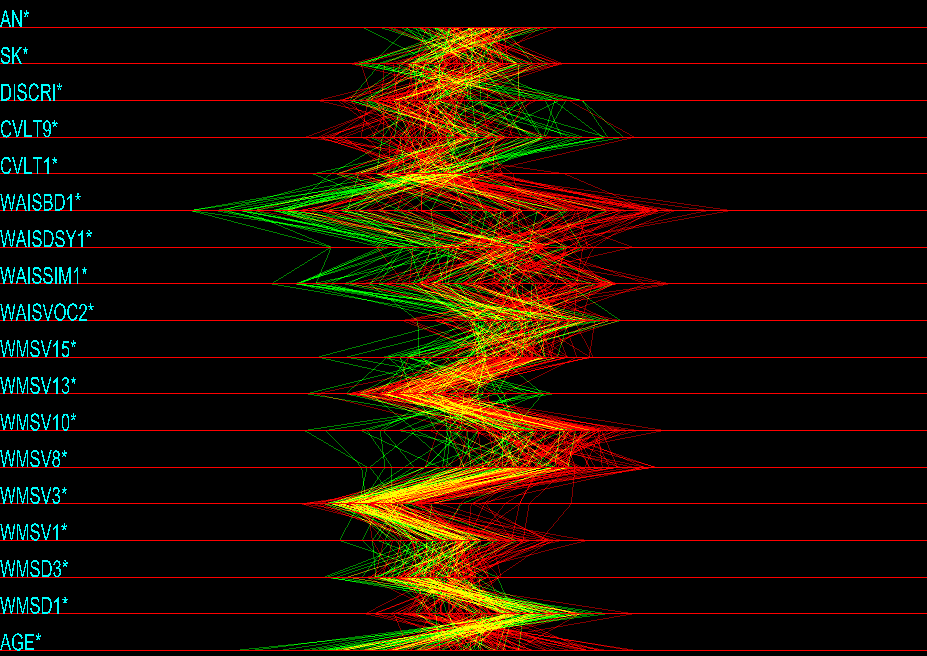

Supplement: Additional file 3 — A parallel coordinate plot of the data after a rotation transformation in the data space. The axes correspond now to linear combinations of the original variables. The 6th axis from the top shows an interesting one-dimensional projection of the data. In this direction the two clusters are well separated. The above figure was produced using the Crystal Vision software. The software uses a so-called grand tour technique to systematically (and in a continous manner) go through all possible rotations in the data space. The tour can be visually monitored using the parallel coordinate plot. [file 1471-244X-4-20-S3.gif]

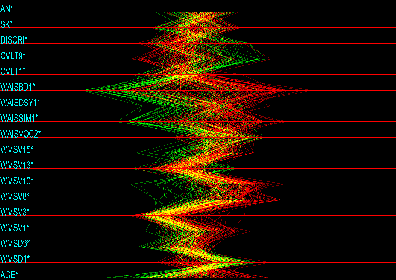

Supplement: Additional file 4 — Smaller version of Additional file 3. A parallel coordinate plot of the data after a rotation transformation in the data space. The axes correspond now to linear combinations of the original variables. The 6th axis from the top shows an interesting one-dimensional projection of the data. In this direction the two clusters are well separated. The above figure was produced using the Crystal Vision software. The software uses a so-called grand tour technique to systematically (and in a continous manner) go through all possible rotations in the data space. The tour can be visually monitored using the parallel coordinate plot. [file 1471-244X-4-20-S4.gif]
